# Performance and impact of a multiplex PCR in ICU patients with ventilator-associated pneumonia or ventilated hospital-acquired pneumonia

**DOI:** 10.1186/s13054-020-03067-2

**Published:** 2020-06-19

**Authors:** Nathan Peiffer-Smadja, Lila Bouadma, Vincent Mathy, Kahina Allouche, Juliette Patrier, Martin Reboul, Philippe Montravers, Jean-François Timsit, Laurence Armand-Lefevre

**Affiliations:** 1 Université de Paris, IAME, INSERM, Paris, F-75018 France; 2 Infectious and Tropical Diseases Department, Bichat-Claude Bernard Hospital, AP-HP, Paris, 75018 France; 3 Medical and Infectious Diseases ICU (MI2), Bichat-Claude Bernard Hospital, AP-HP, 75018 Paris, France; 4 Bacteriology Laboratory, Bichat-Claude Bernard Hospital, AP-HP, Paris, France; 5 Département d’Anesthésie Réanimation, Bichat-Claude Bernard Hospital, AP-HP, Paris, France; 6 INSERM UMR 1152, Physiopathologie et Epidémiologie des Maladies respiratoires, Paris, France

**Keywords:** Multiplex PCR, Hospital-acquired pneumonia, Ventilator-associated pneumonia, Rapid diagnostics, Antibiotic stewardship, Antimicrobial resistance, Point-of-care testing

## Abstract

**Background:**

Early appropriate antibiotic therapy reduces morbidity and mortality of severe pneumonia. However, the emergence of bacterial resistance requires the earliest use of antibiotics with the narrowest possible spectrum. The Unyvero Hospitalized Pneumonia (HPN, Curetis) test is a multiplex PCR (M-PCR) system detecting 21 bacteria and 19 resistance genes on respiratory samples within 5 h. We assessed the performance and the potential impact of the M-PCR on the antibiotic therapy of ICU patients.

**Methods:**

In this prospective study, we performed a M-PCR on bronchoalveolar lavage (BAL) or plugged telescoping catheter (PTC) samples of patients with ventilated HAP or VAP with Gram-negative bacilli or clustered Gram-positive cocci. This study was conducted in 3 ICUs in a French academic hospital: the medical and infectious diseases ICU, the surgical ICU, and the cardio-surgical ICU. A multidisciplinary expert panel simulated the antibiotic changes they would have made if the M-PCR results had been available.

**Results:**

We analyzed 95 clinical samples of ventilated HAP or VAP (72 BAL and 23 PTC) from 85 patients (62 males, median age 64 years). The median turnaround time of the M-PCR was 4.6 h (IQR 4.4–5). A total of 90/112 bacteria were detected by the M-PCR system with a global sensitivity of 80% (95% CI, 73–88%) and specificity of 99% (95% CI 99–100). The sensitivity was better for Gram-negative bacteria (90%) than for Gram-positive cocci (62%) (*p* = 0.005). Moreover, 5/8 extended-spectrum beta-lactamases (CTX-M gene) and 4/4 carbapenemases genes (3 NDM, one oxa-48) were detected. The M-PCR could have led to the earlier initiation of an effective antibiotic in 20/95 patients (21%) and to early de-escalation in 37 patients (39%) but could also have led to one (1%) inadequate antimicrobial therapy. Among 17 empiric antibiotic treatments with carbapenems, 10 could have been de-escalated in the following hours according to the M-PCR results. The M-PCR also led to 2 unexpected diagnosis of severe legionellosis confirmed by culture methods.

**Conclusions:**

Our results suggest that the use of a M-PCR system for respiratory samples of patients with VAP and ventilated HAP could improve empirical antimicrobial therapy and reduce the use of broad-spectrum antibiotics.

## Introduction

Hospital-acquired pneumonia (HAP) and ventilator-associated pneumonia (VAP) are the most common healthcare-associated infections in adults and are the leading causes of death in critical care [1, 2]. HAP and VAP are associated with a longer duration of mechanical ventilation, ICU stay, hospitalization, and increased healthcare cost [3]. They are thus associated with an excess of morbidity and mortality [4]. HAP and VAP may be caused by a wide variety of pathogens and can be polymicrobial [5, 6]. Moreover, multidrug-resistant (MDR) and extensively drug-resistant (XDR) bacteria, especially Gram-negative bacilli, are increasingly frequently isolated in HAP and VAP and are associated with mortality rates over 50% [7]. Early appropriate antibiotic therapy undoubtedly reduces morbidity and mortality of HAP and VAP, but these infections are responsible for up to half of the consumption of antibiotics in ICU [8, 9]. Therefore, international guidelines advocate the empirical use of broad-spectrum antibiotics including carbapenems in the treatment of VAP caused by Gram-negative bacilli in the case of prior antibiotic therapy, in patients colonized by multidrug-resistant bacteria (MDR), or in any late-onset VAP (more than 5 days after the beginning of mechanical ventilation) [1, 4]. Usually, the time between respiratory sampling and the definitive microbiological results including antibiotic susceptibility testing is at least 48 h, during which time treatment is empirical. However, even very short exposure (1 to 3 days) to carbapenem is associated with a 5-fold higher risk of emergence of imipenem-resistant Gram-negative bacteria in the intestinal microbiota of ICU patients [10]. Intensivists are thus confronted with a permanent dilemma between the initiation of adequate antibiotic therapy and the risk of increasing MDR bacteria [3, 11] by the prescription of broad-spectrum antibiotics.

The development and use of rapid, effective, and inexpensive diagnostic tests to accelerate the classical process of microbiological diagnosis are among the most important actions to fight against antimicrobial resistance. Rapid microbiological results of respiratory samples in suspected VAP and HAP could allow the rapid use of narrow-spectrum antibiotics [12]. New rapid diagnostic tests, based on molecular methods directly performed on clinical samples, are emerging in the diagnostic market [13]. They allow the identification of the micro-organisms present in the clinical sample and the detection of targeted resistance genes only a few hours after sampling. The Unyvero platform (Curetis AG, Holzgerlingen, Germany) is a multiplex PCR (M-PCR) rapid diagnosis system that can be used for the microbiological diagnosis of severe infections within 5 h [14]. The Unyvero HPN (hospitalized pneumonia) M-PCR panel detects 21 bacteria involved in community and hospitalized acquired pneumonia, including most of the *Enterobacterales*, *Pseudomonas aeruginosa*, *Stenotrophomonas maltophilia*, or *Staphylococcus aureus*, as well as 21 antibiotic resistance genes such as the most common extended-spectrum beta-lactamases (ESBL), carbapenemases genes and *mecA* gene (Supplementary material).

However, few data are available on the performance of M-PCR for microbiological diagnosis in real-life and even less on their impact on antibiotic use and clinical outcomes. Moreover, the optimal place of M-PCR in the diagnostics process still has to be determined. In this study, we evaluated the diagnostic performance of the Unyvero HPN test compared to standard microbiological tests and the potential impact of its results on early adaptation of antimicrobial therapy in ICU patients with suspected ventilated HAP or VAP.

## Material and methods

### Study design

This is a prospective study, performed between May 2017 and November 2018 in the 3 ICUs of Bichat-Claude Bernard University Hospital (Paris, France): the 25-bed medical and infectious diseases ICU, the 17-bed surgical ICU, and the 15-bed cardio-surgical ICU.

### Patient selection

We selected patients with a ventilated HAP or a suspicion of VAP who had a bronchoalveolar lavage (BAL) or plugged telescoping catheter (PTC) sample with Gram-negative bacilli or clustered Gram-positive cocci on Gram staining. Pneumonia were diagnosed according to the IDSA guidelines for VAP: new lung infiltrate on a chest X-ray and evidence that the infiltrate was of an infectious origin, i.e., new onset of fever (> 38.5 °C), and/or purulent sputum, and/or leukocytosis, and/or decline in oxygenation [1].

### Microbiological performance

**Fig. 1:**
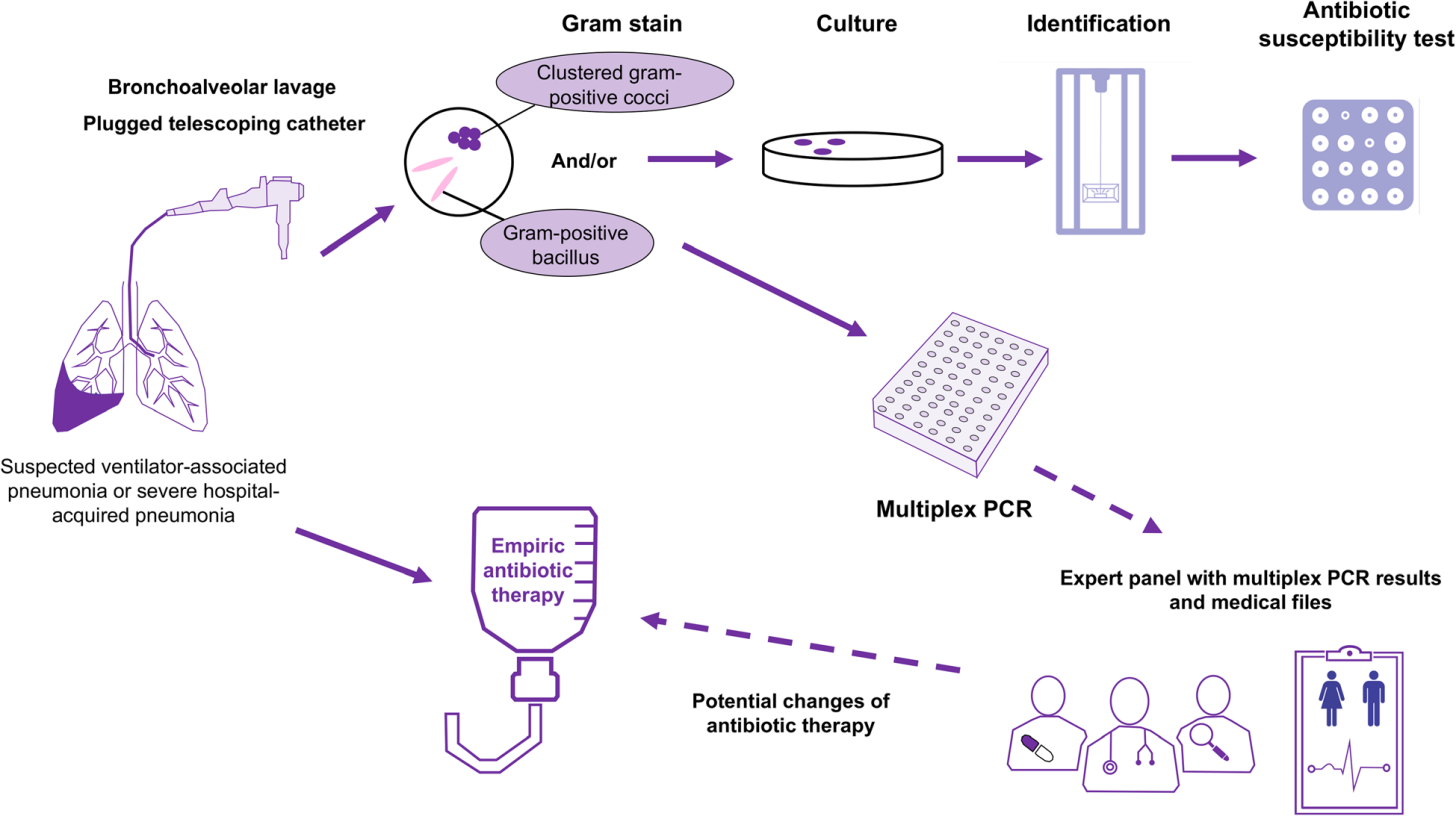
Visual summary of the study

The respiratory samples were analyzed using conventional microbiological methods including quantitative culture, bacterial identifications performed by MALDI-TOF mass spectrometry, and antibiotic susceptibility testing performed by disk diffusion method according to EUCAST recommendations. We performed a Unyvero HPN test on every sample and evaluated the performance of the test compared to conventional microbiological methods (i) considering micro-organisms that reached clinical thresholds (10^4^ colony-forming unit (CFU)/ml for BAL and 10^3^ CFU/ml for PTC) and (ii) considering all micro-organisms identified in culture. We defined discordance whenever the M-PCR detected an organism that was not detected by culture (false positive) or the culture detected an organism that was not detected by multiplex PCR (false negative). Regarding resistance genes, we reported the results for the following resistance genes: *mecA*, *mecC* (methicillin resistance), *bla*_OXA-23_, *bla*_OXA-24_, *bla*_OXA-48_, *bla*_OXA-58_*bla*_VIM_, *bla*_IMP_, *bla*_KPC_, *bla*_NDM_ (carbapenemases), and *bla*_CTX-M_ (ESBL). The turnaround time of the M-PCR was reported as the time from placing the sample in the M-PCR system to the final results (Fig. 1).

### Potential impact on antibiotic therapy

A multidisciplinary group including intensivists and clinical microbiologists simulated the antibiotics changes they would have made to the empiric antibiotic therapy if the M-PCR results had been available on the day of the sampling. The antimicrobial selection in our centre follows the ATS/IDSA guidelines [1] adapted to the local epidemiology of antimicrobial resistance [15].

The group successively reviewed all the data without and then with the results of the M-PCR. The data reviewed were the entire medical file of the patients including clinical, radiological, and Gram staining results related to the episode of pneumonia and medical history with comorbidities as well as previous bacteriological results including carriage, colonization, and infections with susceptibility profiles and previous antimicrobial therapies. Antibiotic changes were split into appropriate and inappropriate changes. Appropriate changes included adequacy, de-escalation, and optimization of the antibiotic therapy, and inappropriate changes included inadequacy, escalation, and de-optimization. We defined adequacy as the introduction of an effective antibiotic on causative bacteria that were not correctly treated before the results of the M-PCR. We defined de-escalation as the appropriate use of a narrower-spectrum antibiotic as described by Weiss et al. for β-lactam antibiotics [16]. De-escalation was considered (i) when a carbapenem was replaced by another β-lactam antibiotic; (ii) when piperacillin/tazobactam or a fourth-generation cephalosporin was replaced by amoxicillin, amoxicillin/clavulanic acid, piperacillin, ticarcillin, or a third-generation cephalosporin; and (iii) when a third-generation cephalosporin was replaced by amoxicillin/clavulanic acid or amoxicillin (Supplementary material). In the case of treatment with a combination of β-lactam plus aminoglycoside, only susceptibility to the β-lactam was taken into account because monotherapy with aminoglycosides was considered inadequate. We defined optimization as the use of a fourth-generation cephalosporin such as cefepime instead a third-generation cephalosporin for the treatment of AMP-C producing *Enterobacteriales* (*Enterobacter* spp*.*, *Serratia marcescens*, *Citrobacter freundii*, or *Morganella morganii*) [17]. De-optimization was the opposite. The change was considered as inadequate (inadequacy) when Unyvero results led to a switch from an effective antibiotic to an ineffective antibiotic on causative bacteria. An escalation was the introduction after the results of the M-PCR of an antibiotic with a broader spectrum that was not needed in light of the culture results.

### Data analysis

Data were entered into a spreadsheet and imported into R software (version 3.2.4) for statistical analysis. Numerical data are presented as absolute numbers, proportion, or median ± interquartile range (IQR). We used Pearson’s chi-squared test to compare the sensitivity between subgroups of samples.

### Ethics

The M-PCR was performed on clinical specimens taken as part of routine care and tested in the clinical microbiology laboratory. No additional samples were collected for this study. Data were collected prospectively, anonymously during the study period, in compliance with the GDPR. The Institutional Review Board 00006477 of Paris University, Assistance Publique – Hôpitaux de Paris, authorized the study and waived the need for informed consent. The study complied with the Standards for the Reporting of Diagnostic Accuracy studies recommendations.

## Results

### Demographical characteristics

We analyzed 95 clinical samples, 72 BAL and 23 PTC, from 85 patients, 73% men, median age 64 years (IQR 54–69). Among the 95 episodes of pneumonia, 71 were ventilator-associated and 24 were ventilated hospital-acquired pneumonia. Thirty-one patients were immunocompromised: ten were heart transplant recipients, ten lung transplant recipients, six had chemotherapy for cancer, and five had acquired immune deficiency syndrome (AIDS). The median SAPS II score was 59 (IQR 36–72), and the mortality rate was 32% during the stay in intensive care. Antibiotics had been prescribed in the 7 days before the diagnosis of pneumonia in 41 episodes (43%), including cefotaxime (12%), piperacillin-tazobactam (7%), amikacin (7%), and cefepime (6%) among the most frequently prescribed antibiotics. Patient characteristics are reported in Table 1.

**Table 1:** Demographics and baseline clinical characteristics of the patients and clinical samples

Patients	Number (%) (*n* = 85)	Median [IQR]
Age (years)		64 [54–69]
Male	62 (73)	
Co-morbid conditions		
Heart transplant	10 (12)	
Lung transplant	10 (12)	
COPD	14 (16)	
Active chemotherapy for cancer*	6 (7)	
AIDS	5 (6)	
Chronic dialysis	3 (4)	
Clinical samples	Number (%) (*n* = 95)	Median [IQR]
Pneumonia		
Ventilator-associated	71 (75)	
Ventilated hospital-acquired	24 (25)	
Severity of disease		
Days in intensive care		5.6 [3.5–14]
SAPS II score		59 [36–72]
Days of mechanical ventilation		5.5 [3.4–11]
Relapse of pneumonia	24 (25)	
Deaths	30 (32)	
Laboratory		
ESBL carrier	23 (24)	
MRSA carrier	1 (1)	
Antibiotics in the 7 days before the episode of pneumonia**	41 (43)	
Cefotaxime	11 (12)	
Piperacillin-tazobactam	7 (7)	
Amikacin	7 (7)	
Cefepime	6 (6)	
Metronidazole	5 (5)	
Sampling		
Bronchoalveolar lavage	72 (76)	
Plugged telescoping catheter	23 (24)	
Turnaround time of Unyvero HPN (hours)		4.6 [4.4–5]
Lysis		0.5 [0.5–0.5
Waiting time		0.1 [0.1–0.4]
Analysis		3.8 [3.8–3.8]

*Three patients with lung cancer, 2 with esophagus cancer, and one with melanoma

**Only the 5 most frequently prescribed antibiotics are reported

### Microbiological outcomes

The median turnaround time of the M-PCR was 4.6 h (IQR 4.4–5). Overall, 104 bacteria were identified using the M-PCR and 128 by conventional culture. The most frequently identified bacteria were *Pseudomonas aeruginosa* (*n* = 32 and *n* = 33 respectively on culture and M-PCR), *Escherichia coli* (*n* = 15 on culture and M-PCR), *Klebsiella pneumoniae* (*n* = 14 and *n* = 9), and *Staphylococcus aureus* (*n* = 12 and 8).

When considering the micro-organisms isolated at clinical thresholds (10^4^ CFU/ml for BAL and 10^3^ CFU/ml for PTC), 90/112 bacteria were detected by the M-PCR which yielded a sensitivity of 80% (95% CI, 71–88%), a specificity of 99% (95% CI, 99–100%), a positive predictive value of 87% (95% CI, 80–93%), and a negative predictive value of 99% (95% CI, 99–99%) (Table 2). The sensitivity of the M-PCR was very heterogeneous among bacteria, ranging from 100% for *Pseudomonas aeruginosa* (*n* = 32) or *Proteus* spp. (*n* = 7) to 0% for *Streptococcus pneumoniae* (*n* = 2), 33% for *Morganella morganii* (*n* = 3), 67% for *Enterobacter cloacae* (*n* = 6), or 73% for *Staphylococcus aureus* (*n* = 11). Overall, the sensitivity was better for Gram-negative bacteria (90%) than for Gram-positive cocci (62%) (*p* = 0.005). The sensitivity of the M-PCR was not different between samples performed in patients who had antibiotics in the previous 7 days (*n* = 41, sensitivity of 82%) or patients who did not (*n* = 54, sensitivity of 79%) (*p* = 0.88). Among 29 polymicrobial samples (31%), the M-PCR detected 44/60 bacteria which corresponds to a sensitivity of 73%, compared to an 88% sensitivity for monomicrobial samples (46/52) (*p* = 0.08). The specificity of the M-PCR was excellent, between 97 and 100% for all bacteria isolated at clinical thresholds.

**Table 2:** Performance of multiplex PCR (M-PCR) for the identification of micro-organisms isolated at clinical thresholds

	Organism	True positive (culture = M-PCR)	False positive (M-PCR+/culture −)	False negative (culture+/M-PCR−)	Se (%) [95% CI]	Sp (%) [95% CI]	PPV (%) [95% CI]	NPV (%) [95% CI]
Gram-positive bacteria	*Staphylococcus aureus*	8	0	3	73	100	100	97
	*Streptococcus pneumoniae*	0	0	2	0	100	–	98
*Enterobacteriaceae*	*Citrobacter freundii*	0	0	0	–	100	–	100
	*Escherichia coli*	12	3	1	92	96	80	99
	*Enterobacter cloacae complex*	4	2	2	67	98	67	98
	*Enterobacter aerogenes*	1	0	0	100	100	100	100
	*Proteus* spp.	7	0	0	100	100	100	100
	*Klebsiella pneumoniae*	9	0	3	75	100	100	97
	*Klebsiella oxytoca*	2	2	0	100	98	50	100
	*Klebsiella variicola*	0	1	0	–	99	0	100
	*Serratia marcescens*	5	0	0	100	100	100	100
	*Morganella morganii*	1	1	2	33	99	50	98
	Hafnia alvei*	0	0	5	0	100	–	95
	Citrobacter koseri*	0	0	2	0	100	–	98
	Serratia rubidaea*	0	0	1	0	100	–	99
Non-fermenting bacteria	*Moraxella catarrhalis*	1	1	0	100	99	50	100
	*Pseudomonas aeruginosa*	31	2	0	100	97	94	100
	*Acinetobacter baumannii complex*	3	0	0	100	100	100	100
	*Stenotrophomonas maltophilia*	1	2	0	100	98	33	100
	*Legionella pneumophila*	2	0	0	100	100	100	100
Others	*Pneumocystis jirovecii*	0	0	0	–	100	–	100
	*Haemophilus influenzae*	3	0	1	75	100	100	99
	*Mycoplasma pneumoniae*	0	0	0	–	100	–	100
	*Chlamydophila pneumoniae*	0	0	0	–	100	–	100
Total		90	14	22	80 [73–88]	99 [99–100]	87 [80–93]	99 [99–99]

For culture, only bacteria that were superior to diagnostic thresholds (10^4^ CFU/ml for BAL and 10^3^ CFU/ml for PTC) were considered

*Organisms not screened on the multiplex PCR system

The M-PCR yielded 14 false positive results: 3 *Escherichia coli*, 2 *Enterobacter cloacae*, 2 *Klebsiella oxytoca*, 2 *Pseudomonas aeruginosa*, 2 *Stenotrophomonas maltophilia*, 1 *Klebsiella variicola*, 1 *Morganella morganii*, and 1 *Moraxella catarrhalis* that were not found in conventional cultures. In 8 pneumonia episodes, causative bacteria were not detected by the M-PCR because of 3 bacterial species that are not included in the M-PCR: *Hafnia alvei* (*n* = 5), *Citrobacter koseri* (*n* = 2), and *Serratia rubidaea* (*n* = 1). Considering all micro-organisms identified in culture irrespective of the clinical thresholds, 95/118 bacteria were identified which yielded a sensitivity of 81% (95% CI, 72–87%) and a specificity of 99% (95% CI, 99–100%) (Supplementary material).

Regarding antibiotic resistance, the M-PCR detected 5 *bla*_CTX-M_ among 8 (63%) ESBL-producing *Enterobacteriaceae* and 4 carbapenemase genes (*bla*_NDM_ and one *bla*_OXA-23_) out of 4 carbapenemase-producing *Enterobacteriaceae* (100%) (Supplementary material). The M-PCR detected the only methicillin-resistant *S. aureus* isolated in conventional culture but had a false positive for another *mecA* gene. We did not identify any other resistance gene in the study.

### Clinical outcomes

According to the expert panel, having the results of the M-PCR in real-time (the day of sampling) could have led to antibiotic changes in 63/95 (66%) episodes of pneumonia (Table 3). Among the changes, the M-PCR could have led to the earlier initiation of an effective antibiotic in 20/95 patients (21%), to early de-escalation in 37 patients (39%), and to optimization in 3 patients (3%). Among 17 empiric antibiotic treatments with carbapenems, 10 could have been de-escalated in the following hours according to the M-PCR results. However, the M-PCR could also have led to three inappropriate antibiotic switches: one inadequacy and 2 de-optimizations. More precisely, in one case, the M-PCR identified a *Pseudomonas aeruginosa* but missed the presence of an ESBL-producing *Enterobacter cloacae* and could have led to a switch from meropenem to ceftazidime. In two cases, the test missed either an *Enterobacter cloacae* or a *Hafnia alvei* and could have led to a switch from cefepime to ceftazidime.

**Table 3:** Potential impact of multiplex PCR on antibiotic therapy

Initial antibiotic therapy (after Gram stain results)	*n*	Appropriate changes			No change	Inappropriate changes		
		Adequacy	De-escalation	Optimization		Inadequacy	Escalation	De-optimization
Carbapenem + others	17	0	10	0	6	1	0	0
Piperacillin-tazobactam ± aminoglycosides	27	2	15	3	7	0	0	0
Fourth-generation cephalosporin ± aminoglycosides	16	1	7	0	6	0	0	2
Third-generation cephalosporin ± aminoglycosides	11	5	2	0	4	0	0	0
Amoxicillin-clavulanate	5	0	1	0	4	0	0	0
Others*	5	2	2	0	1	0	0	0
No treatment	14	10	0	0	4	0	0	0
Total (%)	95	20 (21)	37 (39)	3 (3)	32 (34)	1 (1)	0	2 (2)

Only bacteria that were superior to diagnostic thresholds (10^4^ CFU/ml for BAL and 10^3^ CFU/ml for PTC) were considered

*Others included cefazolin switched to piperacillin-tazobactam (adequacy), addition of levofloxacin + rifampicin for a *L. pneumophila* (adequacy), colistin switched to cefotaxime (de-escalation), vancomycin switched to oxacillin (de-escalation), unchanged cefazolin

**Fig. 2:**
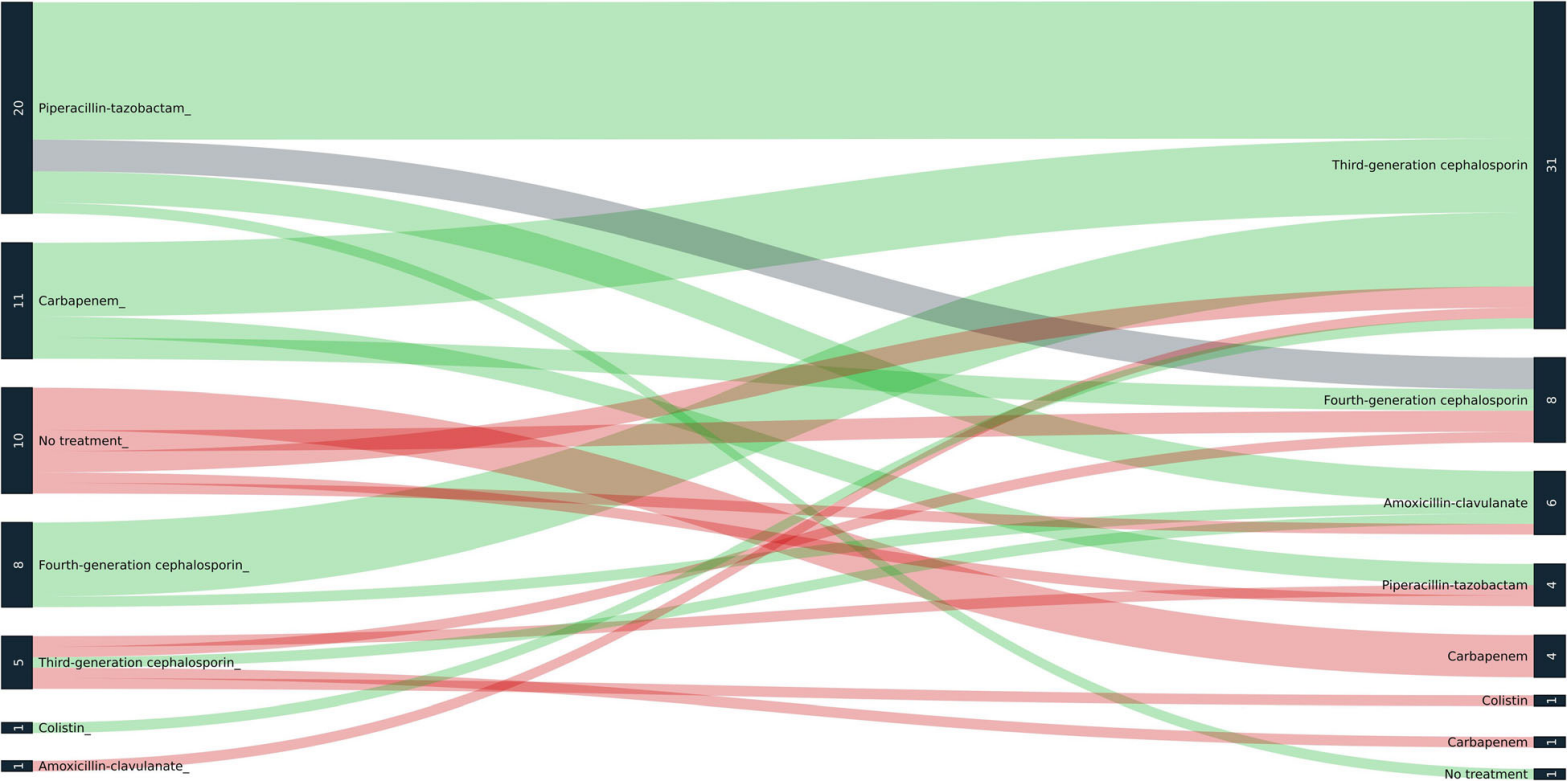
Sankey diagram of potential antibiotic therapy switches following multiplex PCR result. Left: antibiotic therapy following Gram strain results, right: potential antibiotic therapy following multiplex PCR results. The green color is used for antibiotic de-escalation and the red color for antibiotic escalation according to Weiss et al (Supplementary material). The gray color is for switches that are neither escalation nor de-escalation

Figure 2 graphically represents the potential antibiotic changes following the results of the M-PCR. The number of empiric treatments with piperacillin-tazobactam might have decreased from 27 to 10, and the number of treatments with third-generation cephalosporins increased from 11 to 37. The M-PCR also led to 2 unexpected diagnosis of severe legionellosis confirmed by culture methods.

## Discussion

This study, one of the first to analyze the potential impact of a M-PCR system on antimicrobial therapy in the ICU, suggests that it might help improving empiric antibiotic therapy in 63% (60/95) of the patients with suspected VAP or ventilated HAP. The M-PCR provided good overall performances for bacterial identification (sensitivity 80%, 95% CI 71–88%, and specificity 99%, 95% CI 99–100%) and resistance gene detection.

Regarding microbiological performance, our results are very close to similar studies that used the same M-PCR system and reported a sensitivity between 73.1 and 88.8% and a specificity between 94.9 and 97.9% as compared to culture methods [18–20]. As in our study, Papan et al. reported a better performance of the system for Gram-negative bacteria than for Gram-positive cocci on 79 respiratory samples from children and neonates [18]. This might be due to the cell wall structure of Gram-positive bacteria, which have a thicker peptidoglycan than Gram-negative bacteria and thus may be more stable to the lysis process required for DNA extraction.

In our study, the sensitivity of the M-PCR for polymicrobial samples was lower than the overall sensitivity (73% vs 80%) but this difference was not statistically significant. This potential difference probably deserves to be studied in larger samples of patients. We did not encounter machine failure in our study, but machine failure rates over 10% have been reported in the literature [19, 21]. In our study, the M-PCR had a sensitivity of 100% for the detection of carbapenemase genes but only of 63% for the detection of CTX-M genes. This result is difficult to compare with previous studies due to the low number of samples with resistance genes in all studies [18, 22]. The low CTX-M detection rate (63%) observed in our study may be due to the lack of detection of the CTX-M-9 group by the Unyvero HPN kit which only detects the CTX-M-1 group. Indeed, in a recent publication, the proportion of CTX-M-9 group among ESBL-producing *E. coli* isolated in VAP was as high as 40% [23]. This is a limitation of the test for the detection of ESBL that may lead to inappropriate antibiotic prescription.

Obtaining antimicrobial susceptibility results usually takes 48–98 h using conventional methods; thus, the use of molecular diagnostic tools could be helpful to initiate the optimal empiric antibiotic. In this study, a multidisciplinary expert panel reviewed each case and estimated that the M-PCR results would have led to antibiotic changes in 63/95 (66%) patients (60 appropriate changes and 3 inappropriate). It is important to note that we considered a carbapenem as the only effective therapy in patients infected by an ESBL-producing *Enterobacterales*. This is in line with the results of the MERINO trial [24] and with recent studies showing a superiority of carbapenems over piperacillin-tazobactam [25–27]. However, de-escalation to piperacillin-tazobactam could be discussed for stabilized patients infected by bacteria with a MIC under 4 mg/L to piperacillin-tazobactam [28]. Very few studies have assessed the potential impact of a M-PCR system for pneumonia on patient management [29]. In a study on 49 patients with severe HAP, the authors observed that initial empirical treatment was modified in 67.3% of the patients based on the availability of the M-PCR [14]. However, in this study, the authors considered all the pathogens found by the Unyvero system as causative bacteria for the pneumonia and did not report the sensitivity or specificity of the M-PCR as compared to conventional culture. In a case-control study using the BioFire FilmArray (BioMerieux) [30], the authors described an early modification of the antibiotic therapy in 37/56 (66%) patients with severe pneumonia in the ICU.

We identified in our study 3 species of bacteria that are not included in the M-PCR but were involved in 8 pneumonia: *Hafnia alvei* (*n* = 5), *Citrobacter koseri* (*n* = 2), and *Serratia rubidaea* (*n* = 1). The lack of a common marker for *Enterobacteriales* which could detect the presence of any of the species included in this family was considered as a limit by the expert panel. A recent review also underlined the need to include a larger range of targets to improve the sensitivity [31]. It is thus important to note that these tools, because of their lack of exhaustivity, must be used in addition to conventional culture techniques but cannot replace them. M-PCR equipment and cartridge are more costly than conventional methods, and cost-effectiveness studies are needed to decide which samples should be tested with a M-PCR.

The turnaround time of the Unyvero system is longer than the only other M-PCR system currently available for respiratory samples, the BioFire FilmArray (BioMerieux), that allows a diagnosis in 65 min instead of 4–5 h. Moreover, owing to the development of new techniques, M-PCR might become outdated even before they are widely used. Indeed, clinical metagenomics, the comprehensive sequencing of microbial and host genetic material in clinical samples [32], has the potential to improve microbiological diagnostics [33]. In a recent proof of concept study, Langelier et al. combined microbiological and host transcriptome data in tracheal aspirates of 26 patients with a lower respiratory tract infection in the ICU and identified the causative pathogens with an AUC of 0.91 (95% CI, 0.83–0.97); however, this kind of techniques is expensive and not routinely available.

The interpretation and integration of the results into clinical practice required microbiological and antimicrobial expertise, a good knowledge of the performances and limitations of the M-PCR system, and an in-depth discussion between microbiologists and clinicians. Indeed, clinical microbiologists are at the interface between physicians and diagnostic tools and can bridge the gap between demand and supply of innovative systems that could help clinicians take the right decisions at the point of care. It is essential to know the spectrum of the panel to avoid missing treatment for HAP or VAP caused by bacteria that are not included in the panel. The results of this study have to be interpreted in light of the wider context of diagnostics in the ICU: accelerating the microbiology diagnosis is only a part of what is needed to improve empiric antimicrobial therapy [34]. Indeed, the time from sampling to diagnosis is often shorter than the delay between the suspicion of infection and sampling or between microbiological results and change of therapy [35, 36]. Full exploitation of the advantages offered by M-PCR requires a re-organization of the clinical microbiology laboratory, an organized implementation of the technique tailored to the routine workflow, and trained physicians to best integrate the results within clinical care [37].

This study presents several limitations. We simulated the potential impact of the M-PCR results, but clinical studies are obviously needed to evaluate the impact of a M-PCR system in real-life settings. The design of our study does not allow to easily separate what is directly related to the M-PCR results from the impact of a multidisciplinary review of each file. As with other innovative tools [38], clinical trials, preferably randomized and multicentric, should be conducted to evaluate clinical outcomes, including adverse outcomes, process improvement, and ecological impact. Special attention should be paid to the integration and implementation of systems into clinical practice and their adoption and utilization by clinicians. While waiting for the results of clinical trials, implementation outcomes such as appropriateness or fidelity may be key intermediate outcomes to study the success of strategies aiming to bring M-PCR systems to the clinical practice [33]. The question of whether or not M-PCR reduces antibiotic use, antibiotic resistance, direct costs, and indirect costs should be a priority area for future research. In our study, each episode of VAP or ventilated HAP was analyzed by a multidisciplinary expert panel of senior intensivists and clinical microbiologists. In routine practice, this may not always be the case and trials should aim to study the potential difficulties of the integration of M-PCR in the clinical practice such as the management of the results in case of lack of experienced clinical staff to interpret the results. Our results are limited regarding the detection of resistance because of a low number of patients with ESBL or carbapenemase-producing *Enterobacteriaceae* (CPE). Finally, we only included samples with Gram-negative bacilli or clustered Gram-positive cocci on Gram staining in three ICUs in the same hospital preventing the extrapolation of our results to other samples or other contexts. Indeed, the performance of the M-PCR could be different in another setting characterized by a higher proportion of Gram-positive bacteria and/or a different prevalence of *Enterobacterales*.

## Conclusions

Overall, our results suggest that the use of a M-PCR system for VAP and ventilated HAP with good microbiological performances can lead to early adaptation of antimicrobial therapy and thereby limit the selection pressure. Interpretation of the results of the test requires microbiological expertise, an excellent knowledge of the test, and an in-depth discussion between microbiologists and clinicians. These results could inform the development of evidence-based decision algorithms to guide antibiotic prescription and adaptation following M-PCR results in future clinical trials.

## Supplementary information

**Additional file 1.** Micro-organisms tested by Unyvero HPN multiplex PCR system.

**Additional file 2.** De-escalation adapted from the ranking of ß-lactams by Weiss et al.

**Additional file 3.** Analytical performance of the multiplex PCR (M-PCR) with the micro-organisms identified in culture (irrespective of threshold).

**Additional file 4.** Performance of multiplex PCR for the detection of resistance mechanisms.

**Additional file 5.** Detail of the patients with a simulated adequate switch.

## Data Availability

The datasets used and/or analyzed during the current study are available from the corresponding author on reasonable request.
