# Supplementary material for: Performance and impact of a multiplex PCR in ICU patients with ventilator-associated pneumonia or ventilated hospital-acquired pneumonia
Source: Crit Care. 2020 Jun 19;24:366. doi: 10.1186/s13054-020-03067-2 (PMC7303941; doi:10.1186/s13054-020-03067-2)
Supplement: Supplementary file 1 — Additional file 1. Micro-organisms tested by Unyvero HPN multiplex PCR system. [file 13054_2020_3067_MOESM1_ESM.docx]

| Group | Pathogen |  | Gene | Resistance against |
| --- | --- | --- | --- | --- |
| Gram-positive bacteria | *Staphylococcus aureus* |  | *ermB* | Macrolide/Lincosamide |
|  | *Streptococcus pneumoniae* |  | *mecA* | Oxacillin |
| *Enterobacteriaceae* | *Citrobacter freundii* |  | *mecC* (LGA251) | Oxacillin |
|  | *Escherichia coli* |  | *tem* | Penicillin |
|  | *Enterobacter cloacae complex* |  | *shv* | Penicillin |
|  | *Enterobacter aerogenes* |  | *ctx-M* | 3^rd^ generation Cephalosporins |
|  | *Proteus* spp. |  | *kpc* | Carbapenem |
|  | *Klebsiella pneumonia* |  | *imp* | Carbapenem |
|  | *Klebsiella oxytoca* |  | *ndm* | Carbapenem |
|  | *Klebsiella variicola* |  | *oxa-23* | Carbapenem |
|  | *Serratia marcescens* |  | *oxa-24/40* | Carbapenem |
|  | *Morganella morganii* |  | *oxa-48* | Carbapenem |
| Non-fermenting bacteria | *Moraxella catarrhalis* |  | *oxa-58* | Carbapenem |
|  | *Pseudomonas aeruginosa* |  | *vim* | Carbapenem |
|  | *Actinetobacter baumanii complex* |  | *sul1* | Sulfonamide |
|  | *Strenotrophomonas maltophilia* |  | *gyrA83* | Fluoroquinolone |
|  | *Legionella pneumophila* |  | *gyrA87* | Fluoroquinolone |
| Others / Funi | *Pneumocystis jirovecii* |  |  |  |
|  | *Haemophilus influenzae* |  |  |  |
|  | *Mycoplasma pneumoniae* |  |  |  |
|  | *Chlamydophila pneumoniae* |  |  |  |

S1: Micro-organisms tested by Unyvero HPN multiplex PCR system
