# Supplementary figures and images for: Performance and impact of a multiplex PCR in ICU patients with ventilator-associated pneumonia or ventilated hospital-acquired pneumonia
Source: Crit Care. 2020 Jun 19;24:366. doi: 10.1186/s13054-020-03067-2 (PMC7303941; doi:10.1186/s13054-020-03067-2)

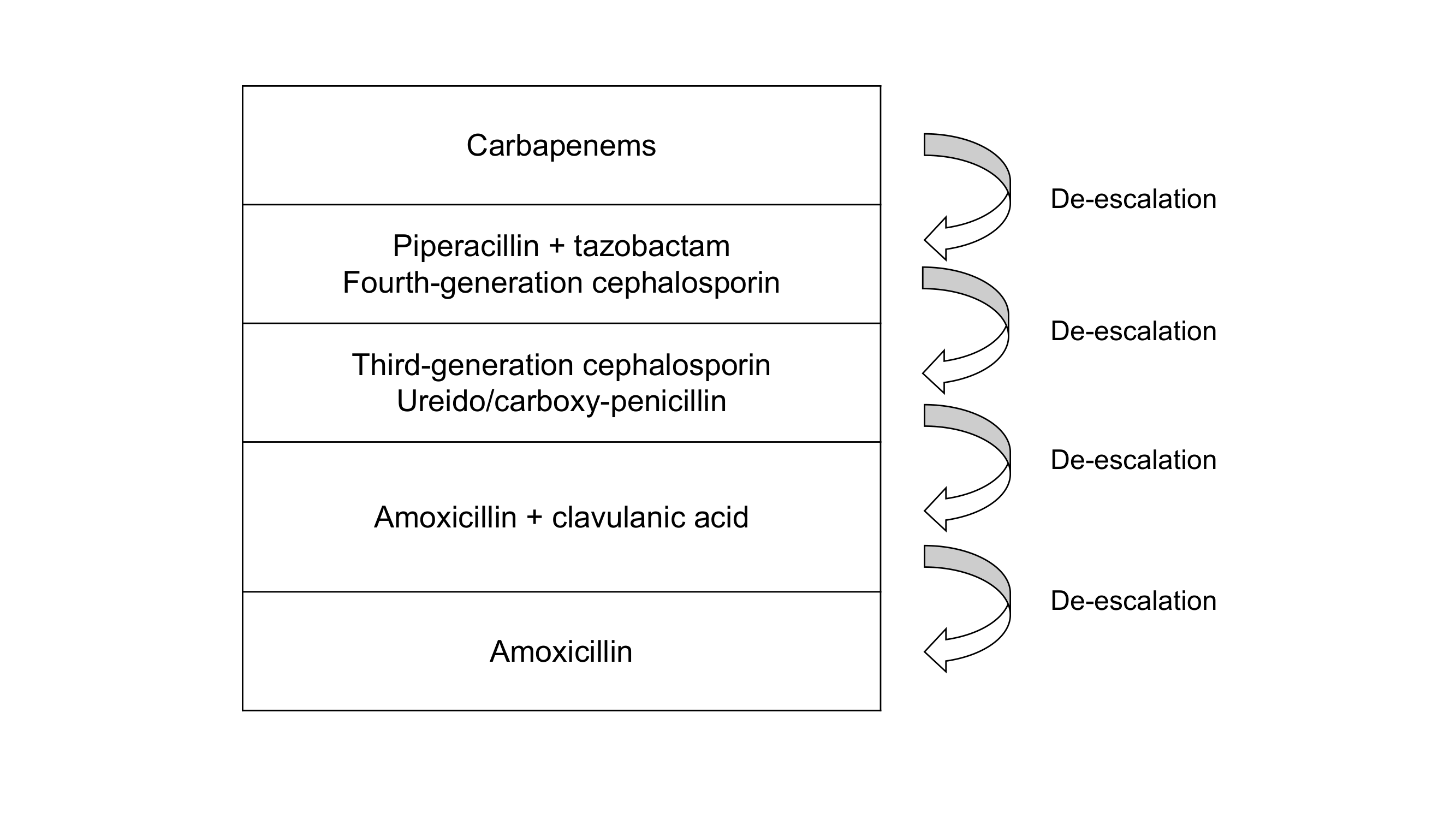

Supplement: Supplementary file 2 — Additional file 2. De-escalation adapted from the ranking of ß-lactams by Weiss et al. [file 13054_2020_3067_MOESM2_ESM.tiff]
