# Supplementary material for: Performance and impact of a multiplex PCR in ICU patients with ventilator-associated pneumonia or ventilated hospital-acquired pneumonia
Source: Crit Care. 2020 Jun 19;24:366. doi: 10.1186/s13054-020-03067-2 (PMC7303941; doi:10.1186/s13054-020-03067-2)
Supplement: Supplementary file 3 — Additional file 3. Analytical performance of the multiplex PCR (M-PCR) with the micro-organisms identified in culture (irrespective of threshold). [file 13054_2020_3067_MOESM3_ESM.docx]

S3: Analytical performance of the multiplex PCR (M-PCR) with the micro-organisms identified in culture (irrespective of threshold)

|  | Organism | True positive  (Culture = M-PCR) | False positive  (M-PCR + / Culture -) | False negative  (Culture + / M-PCR -) | Se (%) [95% CI] | Sp (%)  [95% CI] | PPV (%)  [95% CI] | NPV (%)  [95% CI] |
| --- | --- | --- | --- | --- | --- | --- | --- | --- |
| Gram-positive bacteria | *Staphylococcus aureus* | 8 | 0 | 4 | 67 | 100 | 100 | 95 |
|  | *Streptococcus pneumoniae* | 0 | 0 | 2 | 0 | 100 | - | 98 |
| *Enterobacteriaceae* | *Citrobacter freundii* | 0 | 0 | 0 | - | 100 | - | 100 |
|  | *Escherichia coli* | 14 | 1 | 1 | 93 | 99 | 93 | 99 |
|  | *Enterobacter cloacae complex* | 4 | 2 | 4 | 50 | 98 | 67 | 96 |
|  | *Enterobacter aerogenes* | 1 | 0 | 0 | 100 | 100 | 100 | 100 |
|  | *Proteus* spp. | 7 | 0 | 4 | 64 | 100 | 100 | 95 |
|  | *Klebsiella pneumoniae* | 9 | 0 | 5 | 64 | 100 | 100 | 94 |
|  | *Klebsiella oxytoca* | 2 | 2 | 0 | 100 | 98 | 50 | 100 |
|  | *Klebsiella variicola* | 1 | 0 | 0 | 100 | 100 | 100 | 100 |
|  | *Serratia marcescens* | 5 | 0 | 0 | 100 | 100 | 100 | 100 |
|  | *Morganella morganii* | 2 | 0 | 2 | 50 | 100 | 100 | 98 |
| Non-fermenting bacteria | *Moraxella catarrhalis* | 1 | 1 | 0 | 100 | 99 | 50 | 100 |
|  | *Pseudomonas aeruginosa* | 32 | 1 | 0 | 100 | 98 | 97 | 100 |
|  | *Actinetobacter baumanii complex* | 3 | 0 | 0 | 100 | 100 | 100 | 100 |
|  | *Strenotrophomonas maltophilia* | 1 | 2 | 0 | 100 | 98 | 33 | 100 |
|  | *Legionella pneumophila* | 2 | 0 | 0 | 100 | 100 | 100 | 100 |
| Others | *Pneumocystis jirovecii* | 0 | 0 | 0 | - | 100 | - | 100 |
|  | *Haemophilus influenzae* | 3 | 0 | 1 | 75 | 100 | 100 | 99 |
|  | *Mycoplasma pneumoniae* | 0 | 0 | 0 | - | 100 | - | 100 |
|  | *Chlamydophila pneumoniae* | 0 | 0 | 0 | - | 100 | - | 100 |
| Total |  | 95 | 9 | 23 | 81 [72-87] | 100 [99-100] | 91 [85-95] | 98 [98-99] |
