# Supplementary material for: Performance and impact of a multiplex PCR in ICU patients with ventilator-associated pneumonia or ventilated hospital-acquired pneumonia
Source: Crit Care. 2020 Jun 19;24:366. doi: 10.1186/s13054-020-03067-2 (PMC7303941; doi:10.1186/s13054-020-03067-2)
Supplement: Supplementary file 4 — Additional file 4. Performance of multiplex PCR for the detection of resistance mechanisms. [file 13054_2020_3067_MOESM4_ESM.docx]

S4: Performance of multiplex PCR for the detection of resistance mechanisms

| Group | Resistance | True positive  (Culture = M-PCR) | False positive  (M-PCR + / Culture -) | False negative  (Culture + / M-PCR -) | Se (%) | Sp (%) | PPV (%) | NPV (%) |
| --- | --- | --- | --- | --- | --- | --- | --- | --- |
| Gram-positive bacteria | mecA / mecC | 1 | 1 | 0 | 100 | 86 | 50 | 100 |
| *Enterobacteriaceae* | CTX-M | 5 | 0 | 3 | 63 | 100 | 100 | 96 |
|  | KPC | - |  |  |  |  |  |  |
|  | IMP | - | - | - | - | - | - | - |
|  | NDM | 3 | 0 | 0 | 100 | 100 | 100 | 100 |
|  | VIM | - | - | - | - | - | - | - |
|  | Oxa-48 | - | - | - | - | - | - | - |
| Non-fermenting bacteria | Oxa-23 | 1 | 0 | 0 | 100 | 100 | 100 | 100 |
|  | Oxa-24 | - | - | - | - | - | - | - |
|  | Oxa-58 | - | - | - | - | - | - | - |
| Total |  | 10 | 1 | 3 | 77 [54-100] | 99 [98-100] | 91 [74-100] | 98 [96-100] |
