# Supplementary material for: Performance and impact of a multiplex PCR in ICU patients with ventilator-associated pneumonia or ventilated hospital-acquired pneumonia
Source: Crit Care. 2020 Jun 19;24:366. doi: 10.1186/s13054-020-03067-2 (PMC7303941; doi:10.1186/s13054-020-03067-2)
Supplement: Supplementary file 5 — Additional file 5. Detail of the patients with a simulated adequate switch. [file 13054_2020_3067_MOESM5_ESM.docx]

| **Number of the patient** | **Direct smear examination** | **Antibiotic after direct smear examination** | **HPN Unyvero test results** | **Antibiotic simulated after Unyvero HPN test results** | **Culture results** | **Comments simulated antibiotic therapy with Unyvero** |
| --- | --- | --- | --- | --- | --- | --- |
| 1 | some GNB* | piperacillin/tazobactam  ciprofloxacin | *E. coli* | cefotaxime | *E. coli* | Simulated switch with cefotaxime because of *E. coli*. Oxacillinase producing *E. coli*, resistant to piperacillin + tazobactam. |
| 2 | many GNB  few GPC | piperacillin ciprofloxacin | *P. aeruginosa*  *E. coli*  *K. pneumoniae* | ceftazidime amikacin | *P. aeruginosa*  *E. coli*  *K. pneumoniae* | Piperacillin initially started because of a previous colonization with a wild type *P. aeruginosa.* Simulated switch with *c*eftazidime for a *K. pneumoniae* (naturally resistant to piperacillin). |
| 3 | some GNB | cefepime  amikacin | *S. marcescens* | cefepime  ciprofloxacin | *S. marcescens* | Simulated switch with cefepime + ciprofloxacin. Relapse VAP due to *S. marcescens* with intermediary susceptibility to cefepime and resistance to aminoglycosides. |
| 4 | some GNB | ceftazidime  amikacin | *K. pneumoniae* (ESBL)  *P. aeruginosa*  *S. maltophilia* | ceftazidime/avibactam  colimycin  co-trimoxazole | *K. pneumoniae* (CTX-M)  *P. aeruginosa* | Ceftazidime started because of a previous infection with *P. aeruginosa*. Simulated switch ceftazidime/avibactam for an ESBL-*K. pneumoniae* resistant to ceftazidime. Colimycin and co-trimoxazole added but possible false positive *S. maltophilia* |
| 5 | many GNB | ceftazidime  amikacin | *P. aeruginosa* | meropenem  ciprofloxacin | *P. aeruginosa* | Simulated switch for meropenem because of a previous *P. aeruginosa* infection treated with ceftazidime. *P. aeruginosa* resistant to ceftazidime. |
| 6 | many GNB  very few GPC* | cefotaxime | *P. aeruginosa*  *E. cloacae*  *E. aerogenes* | cefepime  amikacin | *P. aeruginosa*  *E. cloacae*  *E. aerogenes* | Simulated switch for cefepime + amikacin because of a *P. aeruginosa* (resistant to cefotaxime) and AmpC producing Enterobacteria |
| 7 | many GPC | cefotaxime | *Proteus spp.*  *M. catarrhalis*  *P. aeruginosa* | piperacillin/tazobactam | *P. mirabilis*  *S. pneumoniae* | Simulated switch with piperacillin/tazobactam for a *P. aeruginosa and a Proteus spp.* Culture did not find the *P. aeruginosa* but the patient had a BAL with >10^7 *P. aeruginosa* four days later. |
| 8 | many GNB | cefotaxime | *L. pneumophila* | levofloxacin  co-trimoxazole | *L. pneumophila* | Simulated switch for levofloxacin for an unexpected *L. pneumophila.* |
| 9 | many GNB  some GPC | cefazolin  ofloxacin | *P. aeruginosa*  *E. cloacae* | piperacillin/tazobactam  ciprofloxacin | *P. aeruginosa* | Cefazolin + ofloxacin because of a simultaneous *S. aureus* bacteremia*.* Simulated switch for a *P. aeruginosa* resistant to cefazolin and ofloxacin. |
| 10 | some GNB | meropenem  amikacin  vancomycin | *L. pneumophila* | meropenem  levofloxacin  rifampicin | *L. pneumophila* | Broad-spectrum antibiotic therapy because patient hospitalized for 8 days for a metastatic melanoma chemotherapy. Simulated switch for levofloxacin + rifampicin for an unexpected *L. pneumophila* |
| 11 | many GNB  some GPC  some GNC* | no antibiotic† | *S. maltophila* | co-trimoxazole | *S. maltophila*  *S. aureus* | Adequate antibiotic therapy 24 hours earlier. |
| 12 | some GNB | no antibiotic† | *A. baumannii* | meropenem  amikacin | *A. pittii*  *K. pneumoniae* | Adequate antibiotic therapy 24 hours earlier. |
| 13 | many GPC | no antibiotic† | *S. aureus* | co-amoxiclav | *S. aureus* | Adequate antibiotic therapy 24 hours earlier. |
| 14 | some GNB  few GPC | no antibiotic† | *A. baumannii*  *K. pneumoniae* | meropenem  colimycin | *A. baumannii*  *K. pneumoniae* | Adequate antibiotic therapy 24 hours earlier. |
| 15 | many GNB  some GPC  some GNC | no antibiotic† | *E. coli* (CTX-M)  *H. influenzae*  *P. aeruginosa*  *M. morganii* | meropenem | *E. coli* (ESBL)  *H. influenzae* | Adequate antibiotic therapy 24 hours earlier (meropenem because of previous 7-day antibiotic therapy with ertapenem). |
| 16 | some GNB  few GPC | no antibiotic† | *P. aeruginosa*  *S. marcescens* | cefepime  ciprofloxacin | *P. aeruginosa*  *S. marcescens* | Adequate antibiotic therapy 24 hours earlier. |
| 17 | very few GNB | no antibiotic† | *P. aeruginosa* | ceftazidime | *P. aeruginosa*  *H. alvei* | Adequate antibiotic therapy 24 hours earlier. |
| 18 | few GNB | no antibiotic† | *P. aeruginosa* | meropenem  amikacin | *P. aeruginosa*  *K. pneumoniae* | Adequate antibiotic therapy 24 hours earlier (meropenem because previous colonization with *P. aeruginosa* ceftazidime resistant). |
| 19 | many GNB | no antibiotic† | *S. marcescens* | cefepime  amikacin | *S. marcescens*  *K. pneumoniae* | Adequate antibiotic therapy 24 hours earlier. |
| 20 | some GPC | no antibiotic† | *M. catarrhalis*  *E. coli* | cefotaxime | *M. catarrhalis*  *E. coli* | Adequate antibiotic therapy 24 hours earlier. |

*GNB: Gram-Negative Bacilli, GNC: Gram-Negative Cocci, GPC: Gram-Positive Cocci

†Patients without antibiotics at D0 were all treated after the results of the conventional culture.
